# Interactions between Neutrophils, Th17 Cells, and Chemokines during the Initiation of Experimental Model of Multiple Sclerosis

**DOI:** 10.1155/2014/590409

**Published:** 2014-02-19

**Authors:** Dagmara Weronika Wojkowska, Piotr Szpakowski, Dominika Ksiazek-Winiarek, Marcin Leszczynski, Andrzej Glabinski

**Affiliations:** ^1^Department of Neurology, Epileptology and Stroke, Medical University of Lodz, Ulice Zeromskiego 113, 90 549 Lodz, Poland; ^2^Department of Propedeutics of Neurology, Medical University of Lodz, Ulice Zeromskiego 113, 90 549 Lodz, Poland

## Abstract

Experimental autoimmune encephalomyelitis (EAE) is an animal model of multiple sclerosis (MS) in which activated T cell and neutrophil interactions lead to neuroinflammation. In this study the expression of CCR6, CXCR2, and CXCR6 in Th17 cells and neutrophils migrating to the brain during EAE was measured, alongside an evaluation of the production of IL-17, IL-23, CCL-20, and CXCL16 in the brain. Next, inflammatory cell subpopulations accumulating in the brain after intracerebral injections of IL-17 or CXCL1, as well as during modulation of EAE with anti-IL-23R or anti-CXCR2 antibodies, were analyzed. Th17 cells upregulate CXCR2 during the preclinical phase of EAE and a significant migration of these cells to the brain was observed. Neutrophils upregulated CCR6, CXCR2, and CXCR6 during EAE, accumulating in the brain both prior to and during acute EAE attacks. Production of IL-17, IL-23, CCL20, and CXCL16 in the CNS was increased during both preclinical and acute EAE. Intracerebral delivery of CXCL1 stimulated the early accumulation of neutrophils in normal and preclinical EAE brains but reduced the migration of Th17 cells to the brain during the preclinical stage of EAE. Modulation of EAE by anti-IL-23R antibodies ameliorated EAE by decreasing the intracerebral accumulation of Th17 cells.

## 1. Introduction

Multiple sclerosis (MS) a demyelinating disease of the central nervous system (CNS) is often characterized by relapsing acute episodes and in many cases evolves into a progressive chronic neurological deterioration [1]. The most commonly used animal model of MS is experimental autoimmune encephalomyelitis (EAE). The many clinical and histopathological similarities between MS and EAE allow results obtained from this model to be extrapolated to human MS [2, 3].

Immunopathogenesis of MS and EAE, despite of many decades of research, remains unclear. According to the current paradigm effector T cells play a key role in the disease development; after migration to the CNS they may initiate autoimmune inflammation and thus damage myelin. Under normal physiological conditions, the blood-brain barrier (BBB) is formed by dense tight junction (TJ) proteins that seal the space between adjacent brain endothelial cells to form a barrier between the circulating blood and the CNS. The capillary endothelial cells of the BBB are surrounded by a basal lamina, pericytes, and astrocytic end-feet with microglia in close proximity. Physiological and pathological changes in the activity of these glial cell populations may weaken BBB integrity [4]. Endothelial cells of the BBB release multiple inflammatory mediators and express various adhesion molecules such as intercellular and vascular cellular adhesion molecules (ICAM-1, VCAM-1), P- and E-selectins. These membrane proteins are required to anchor leukocytes to the vessel wall and are well-established markers of endothelial dysfunction under inflammatory conditions [5]. Migration of lymphocytes through the brain is usually low, as the endothelial BBB limits their entry into the CNS. In the healthy brain, TJ components such as occludin, ZO-1, claudin-3, and claudin-5 are readily detectable [6]. Disruption of the BBB is a crucial event that may permit the entry of inflammatory cells into the brain, a prerequisite for the formation of MS lesions [4].

Evidence for the role of neutrophils, as well as recently discovered Th17 cells in EAE development, continues to increase [7]. Th17 cells and the cytokine IL-17 that they produce [8] mediate the disruption of BBB [9]. IL-17 enhances the activation of matrix metalloproteinase-3 (MMP-3) and attracts neutrophils to the site of inflammation. Enzymes such as MMPs, proteases, and gelatinases that may be activated by neutrophils participate in BBB disruption. The breakdown of BBB effectively increases neutrophil recruitment further, with increased protease activity subsequently attracting a large number of monocytes and macrophages to the inflammatory regions and leading to sustained myelin and axonal damage [10, 11].

In many studies, chemoattractant cytokines, or chemokines, have drawn a great deal of attention, in particular the CC and CXC ELR(−) group of chemokines which are responsible for the chemotaxis of mononuclear cells, a major component of CNS inflammatory infiltrates. However, the role of CXC ELR(+) chemokines such as CXCL1 and CXCL2, which target mainly neutrophils, has not been thoroughly described. Furthermore, cytokines that participate in Th17 cell differentiation and activation such as IL-23, as well as the chemokines CCL20 or CXCL16 and their receptors CCR6 and CXCR6, are also important mediators of this process [12–14].

The major aim of this study was thus to analyze the interactions between Th17 cells and neutrophils in the pathogenesis of early EAE and to define the role of chemokines and their receptors in this interaction.

## 2. Materials and Methods

### 2.1. Animals

All experiments used 8–12 weeks' old female SJL mice. Animals were housed at the animal facility of the Medical University of Lodz, Lodz, Poland, under standard conditions. Experimental protocols were approved by the Animal Care Committee of the Medical University of Lodz.

### 2.2. EAE Induction and Tissue Collection

EAE was induced by active immunization with an encephalitogenic PLP (proteolipid protein) peptide representing residues 139–151 (PLPp: 139–151, Metabion, Martinsried, Germany) emulsified with complete Freund's adjuvant (Sigma, Poznan, Poland). Pertussis toxin (Sigma, Poznan, Poland) was administered by intravenous injection on the day of immunization and again 48 h later, as previously described [2]. Animals were weighed and examined daily for clinical signs of EAE. The following clinical scoring scale was used: 0—no disease symptoms; 1—decreased tail tone or slightly clumsy gait; 2—tail atony and/or moderately clumsy gait and/or poor righting ability; 3—limb weakness; 4—limb paralysis; 5—moribund state [2].

During the preclinical phase (at days 7-8 or 10–13 days after immunization prior to any signs of EAE) and during the initial attack of EAE (the first three days of symptoms) mice were anesthetized with a ketamine/xylazine cocktail (Biowet, Pulawy, Poland) administered intraperitoneally and perfused through the left cardiac ventricle with the ice cold PBS (phosphate buffered saline) (Biomed, Krakow, Poland) containing heparin. Brains, spinal cords, and blood were collected (see below). As a control healthy, nonimmunized mice were used.

### 2.3. Isolation of Mononuclear Cells from the Blood and CNS

Mononuclear cells were isolated from the brain, spinal cord, and blood of immunized mice during the preclinical phase (7-8 days postimmunization; 6 mice) and during the acute attack of the disease (3-4 days after the onset of EAE symptoms; 5 mice). Cells isolated from healthy mice were used as a control (4 mice). Samples were collected from mice by cardiac blood draw using a syringe with heparin. Hematocytes were lysed for 5 min at 4°C with red blood cell lysing buffer (Sigma, Poznan, Poland). The remaining cells were isolated by centrifugation for 10 min at 400 × g (4°C) and then resuspended in PBS.

The CNS (combined brain and spinal cord) collected from animals was placed in the ice cold PBS and forced through 70 *μ*m cell strainers (BD Bioscience, Bedford, MA, USA) to obtain single cell suspensions which were then centrifuged for 10 min at 350 × g (4°C). The CNS mononuclear cells were resuspended in 40% Percoll (Sigma, Poznan, Poland) diluted with white Hank's Buffered Salt Solution (HBSS) (Lonza, Basel, Switzerland). The suspension of cells was then carefully layered on top of the 70% Percoll diluted with red HBSS. CNS mononuclear cells were isolated by centrifugation for 40 min at 700 × g (4°C) with a slow deceleration with no brake. Cells were then collected from the 40%/70% interphase, washed, and resuspended in PBS. Cells suspensions were stained using trypan blue, counted in a Bürker chamber under a light microscope, and prepared for the flow cytometry.

### 2.4. Flow Cytometry Analysis

Single cell suspensions (10^6^ cells) were prepared from CNS and blood and stained with fluorochrome-conjugated antibodies. All monoclonal antibodies (mAb) were purchased from BD Bioscience (Bedford, MA, USA), eBioscience (San Diego, CA, USA), and BioLegend (San Diego, CA, USA). Antibodies were directly labeled with one of the following fluorescent tags: FITC, PE, PerCP, APC, Alexa Fluor 700, and APC-Cy7. Antibodies to the following proteins were used: CD4, CD3, CD11b, CD11c, CD19, CD45, CD14, IL17, CCR6, CXCR6, CXCR2, and Gr-1. Flow cytometry was performed using a BD LSR II flow cytometer and analyzed with BD Diva software. Isotype-matched negative control mouse raised antibodies were used for all stains.

### 2.5. Analysis of Cytokine Level by ELISA

Study groups included 10 to 23 mice divided between the preclinical phase (11–13 days after immunization), acute EAE attack (1–3 days of disease signs), and normal healthy mice as a control. Previously collected brains were homogenized in HEPES buffer with protease inhibitors using a homogenizer (Ultra-Turrax T8, Staufen, Germany). Samples were then centrifuged, supernatants collected, and properly diluted. To estimate the levels of IL-17, IL-23, CCL20, and CXCL16 the Quantikine kit was used as per manufacturer's instructions (R&D Systems, MN, USA).

### 2.6. Stereotactic Brain Microinjections

Stereotactic microinjections were conducted on ketamine/xylazine anesthetized mice on the 4th day after immunization (preclinical phase). Animals were given IL-17, CXCL1, or PBS (control group). The procedure was performed on stereotactic frame (David Kopf Instruments, Tujunga, CA) using a Hamilton syringe (32 G needle, 0.25 mm). Injections were made into the striatum of the brain (in maximal volume of 0.1 *μ*L), which did not cause any apparent neurological impairment in the animals. After intracerebral cytokine administration, the scalp was sutured with surgical thread. Mice were sacrificed 24 h or 72 h after injection of IL-17 and CXCL1 for further analysis. Brains from animals were collected and cells for cytometric analysis were prepared as described above.

### 2.7. Modulation of the Course of EAE and Its Pathology Using Anti-IL23R and Anti-CXCR2 Antibodies

Immunized mice received anti-IL-23R monoclonal, blocking antibody (4 mice), anti-CXCR2 monoclonal, blocking antibody (4 mice) or PBS (control) (4 mice) on the 3rd and 6th day after immunization. Antibodies (at a concentration 20 *μ*g/100 *μ*L) were injected into the tail vein. All mice were weighed and examined daily for clinical signs of EAE. On the second day of the disease mice were anesthetized with a ketamine/xylazine cocktail and perfused with ice cold PBS. Brains were collected from animals and single cell suspensions (10^6^ cells) were prepared. Cells were stained with fluorochrome-conjugated antibodies and a percentage of neutrophils and Th17 cells were analyzed by flow cytometry. Evans blue (EB) at a concentration of 50 mg/mL was administered intraperitoneally on the 2nd day of EAE to experimental and control mice (0.01 mL/g body weight). After 2 hours mice were anesthetized and perfused as described above. Brains were collected and homogenized (Ultra-Turrax T8, Staufen, Germany) in 1 mL of 50% TCA (trichloroacetic acid), centrifuged for 20 min at 10 000 rpm. Then supernatants were collected and diluted 1 : 3 with ethanol. Differences in BBB permeability were measured colorimetrically at a wavelength of 620 nm.

### 2.8. Statistical Analysis

For statistical analysis nonparametric *U* Mann-Whitney tests were used. A value of *P* < 0.05 was considered statistically significant. Data were shown as mean ± SEM.

## 3. Results

### 3.1. Expression of CCR6, CXCR2, and CXCR6 on Th17 Cells and Neutrophils from the CNS and Blood of Mice with EAE

**Figure 1:**
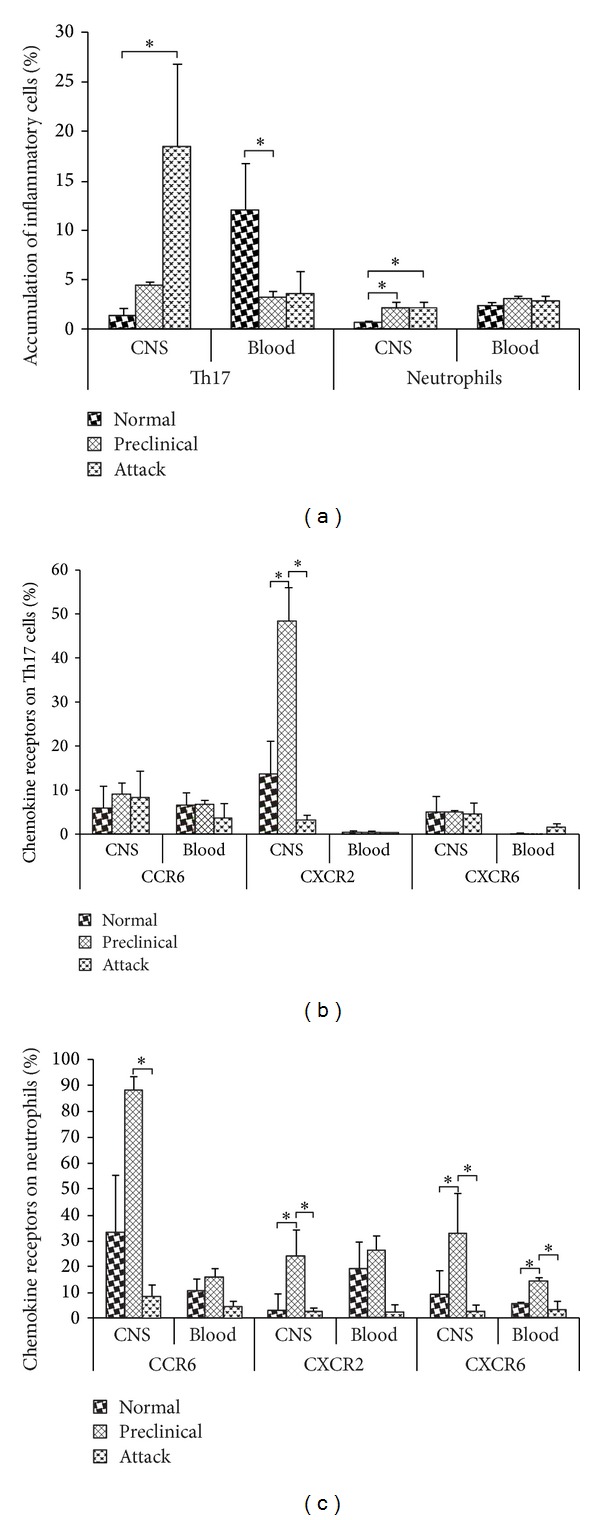
Flow cytometry analysis of chemokine receptors on Th17 cells and neutrophils from the CNS and blood of EAE mice. (a) Percentage of Th17 cell and neutrophils, (b) chemokine receptor expression on Th17 cells, and (c) chemokine receptor expression on neutrophils. Normal—healthy control mice, preclinical—immunized mice before disease onset, attack—second day of EAE symptoms. Data are presented as mean ± SEM. *0.05 ≥ *P* ≥ 0.01.

A statistically significant increase in the number of Th17 cells in samples derived from the brains of mice with EAE attack was observed compared to control animals (*P* value = 0.03). However, the number of Th17 cells in blood during the preclinical phase of EAE was significantly lower when compared to healthy controls (*P* value = 0.02) (Figure 1(a)). During both the preclinical phase and attack of EAE, an increased number of neutrophils was also detected in the CNS in comparison to healthy mice (*P* value = 0.02; *P* value = 0.05, resp.) (Figure 1(a)).

CCR6 expression was present on Th17 cells, but expression levels remained constant regardless of the stage of the disease (Figure 1(b)). A significant increase of CXCR2 expression on Th17 cells was detected in the CNS during the preclinical phase in comparison to healthy mice (*P* value = 0.05), but this expression decreased to control levels in EAE mice (*P* value = 0.02) (Figure 1(b)). Numbers of CXCR6+Th17 cells were on a similar level in all analyzed groups (Figure 1(b)).

CCR6 expression on neutrophils from the CNS was increased in the preclinical phase compared to healthy controls but did not reach statistical significance. A significant decrease of CCR6 expression was, however, observed in the CNS of mice during EAE attack compared to the preclinical phase (*P* value = 0.05) (Figure 1(c)). Significant increases of CXCR2 expression on neutrophils were observed in the CNS during the preclinical phase (*P* value = 0.03), but this expression returned to control levels during EAE attack (*P* value = 0.04) (Figure 1(c)). CXCR6 expression on neutrophils from the CNS was also significantly elevated during the preclinical phase of EAE both when compared to healthy mice and in mice undergoing an EAE attack (*P* value = 0.04; *P* value = 0.04, resp.) (Figure 1(c)). Similarly, there was a significant increase of CXCR6 expression in the blood during the preclinical phase of EAE in comparison to controls and to EAE attack derived samples (*P* value = 0.05; *P* value = 0.02, resp.) (Figure 1(c)).

### 3.2. Production of IL-17, CXCL16, CCL20, and IL-23 in the CNS of Mice with EAE

**Figure 2:**
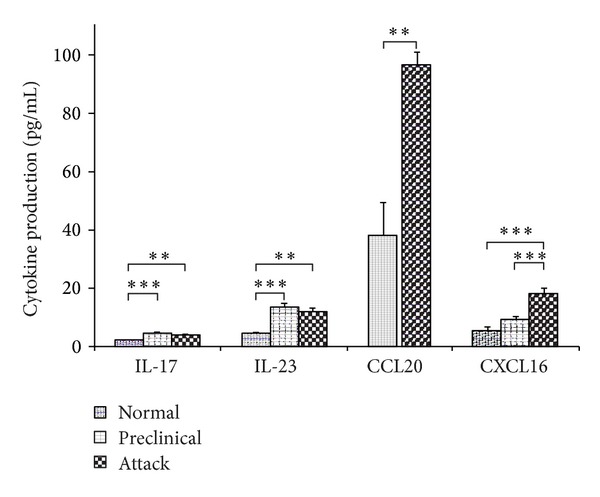
Cytokine production in the brain of EAE mice. Normal—healthy control mice, preclinical—immunized mice before disease onset, attack—second day of EAE symptoms. Data are presented as mean ± SEM. *0.05 ≥ *P* ≥ 0.01; **0.01 > *P* ≥ 0.005; ***P < 0.005.

A significant increase of IL-17 production was observed in the brains of mice in the preclinical phase of EAE (*P* value = 0.0003) as well as during the initial attack of the disease (*P* value = 0.005) when compared to controls (Figure 2). A significant increase of IL-23 levels was also observed in brains collected during the preclinical phase of EAE (*P* value = 0.003) and the EAE attack compared to normal brains (*P* value = 0.009) (Figure 2). Similarly, analysis of CCL20 concentration in the CNS showed a significant increase during EAE attack in comparison to healthy controls (*P* value = 0.006) (Figure 2). Measures of CXCL16 concentrations in brain showed significant differences between both mice undergoing an acute EAE attack (*P* value = 0.000008) and mice in the preclinical phase (*P*-value = 0.002), compared to normal controls (Figure 2).

### 3.3. Accumulation of Neutrophils and Th17 Cells in the Brain after Intracerebral Injection of IL-17 or CXCL1

**Figure 3:**
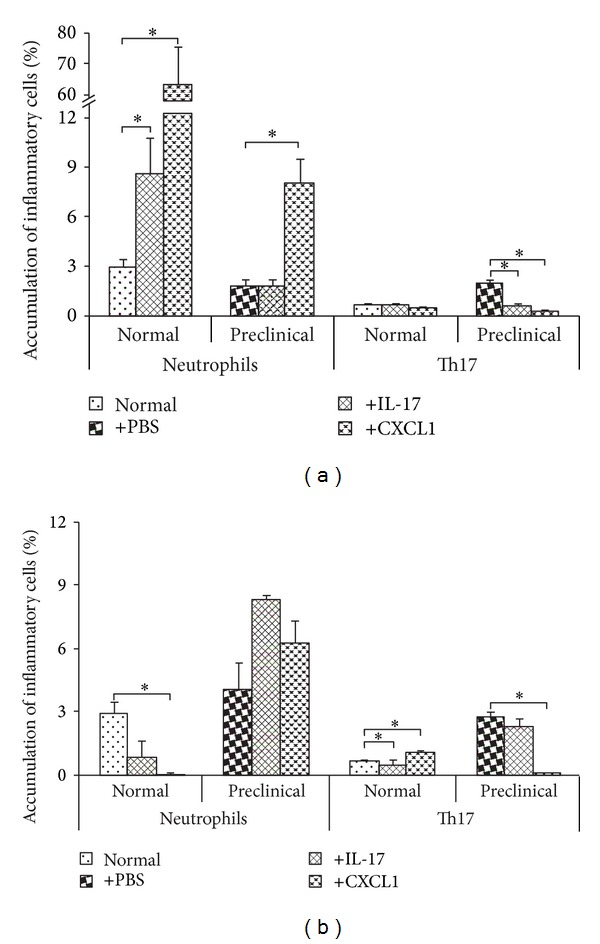
Accumulation of Th17 cells and neutrophils in the brain after stereotaxic intracerebral delivery of IL-17 or CXCL1. (a) 24 h after delivery, (b) 72 h after delivery. Accumulation of cells was measured in healthy control (Normal) mice and in immunized, preclinical mice by flow cytometry. Data are presented as mean ± SEM. *0.05 ≥ *P* ≥ 0.01.

Neutrophil accumulation in the normal brain significantly increased at 24 h after either IL-17or CXCL1 stereotaxic injections (*P* value = 0.02 for both) (Figure 3(a)). Furthermore, neutrophil infiltration of the preclinical EAE brain after CXCL1 administration was higher than in PBS control injected mice (*P* value = 0.05) (Figure 3(a)).

Accumulation of Th17 cells was unchanged 24 h after IL-17 and CXCL1 injections into the normal brain. Interestingly, accumulation of Th17 cells in the brain of immunized, preclinical mice was significantly lower after IL-17 and CXCL1 intracerebral delivery than after control PBS injection (*P* value = 0.049 and *P* value = 0.03, resp.) (Figure 3(a)).

At 72 h after CXCL1 intracerebral injection, the accumulation of neutrophils in the normal brain was significantly lower than in control uninjected brain (*P* value = 0.01) (Figure 3(b)). At this time point, IL-17 or CXCL1 delivery to the preclinical EAE brain did not change the accumulation of neutrophils when compared to control PBS-injected animals (Figure 3(b)).

In normal brain the accumulation of Th17 cells was diminished at 72 h after IL-17 injection but increased after CXCL1 injection when compared to uninjected normal brain (*P* value = 0.02 and *P* value = 0.03, resp.) (Figure 3(b)). At that time point intracerebral CXCL1 delivery significantly reduced the accumulation of Th17 cells in the brain of preclinical mice in comparison to control PBS administration (*P* value = 0.01) (Figure 3(b)).

### 3.4. Modulation of EAE Course and Pathology by Anti-CXCR2 or Anti-IL-23R Antibodies

**Figure 4:**
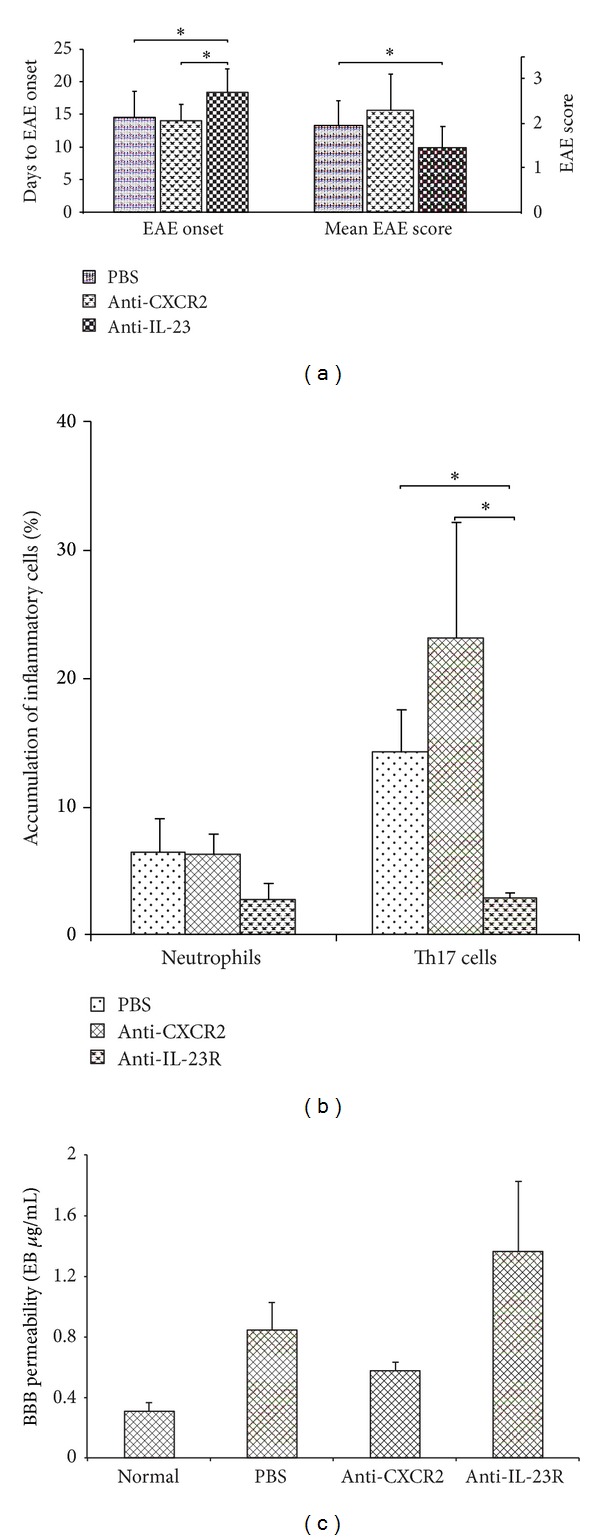
Modulation of EAE by anti-CXCR2 or anti-IL23R antibodies. (a) Mean time to EAE onset and mean EAE score at day 2 of the attack, (b) accumulation of Th17 cells and neutrophils in the brain, and (c) BBB permeability. Accumulation of inflammatory cell subpopulations was analyzed at the second day of EAE using flow cytometry; BBB permeability was analyzed at the same time point using Evans blue dye as described in Materials and Methods. Data are presented as mean ± SEM. *0.05 ≥ *P* ≥ 0.01.

Modulation of EAE with a specific anti-IL-23R monoclonal antibody significantly delayed the appearance of the first clinical symptoms compared to control mice receiving PBS (*P* value = 0.03), as well as to mice treated with anti-CXCR2 monoclonal antibody (*P* value = 0.01) (Figure 4(a)). This treatment, with anti-IL23R antibodies, also significantly reduced the severity of symptoms in this model as measured by EAE score on the second day of the disease (*P* value = 0.05) (Figure 4(a)).

During the second day of the EAE attack, the accumulation of neutrophils in the brain was similar in all analyzed groups. Interestingly, at that time point, the number of Th17 cells in brains of mice treated with anti-IL-23R antibody was significantly lower when compared to control mice (*P* value = 0.02) or mice treated with anti-CXCR2 monoclonal antibody (*P* value = 0.03) (Figure 4(b)).

Also at that time point, the greatest disruption of BBB permeability, as measured by Evans blue accumulation in the brain, was observed in mice treated with anti-IL-23R antibody, but this did not reach statistical significance (Figure 4(c)).

## 4. Discussion

Our study has demonstrated that Th17 cells and neutrophils, as well as inflammatory mediators that they produce, play a very important role in the development of autoimmune CNS inflammation during the early stages of EAE. Activated Th17 cells are the major producers of the inflammatory cytokine IL-17 [15], which is known to be produced also by neutrophils [16]. In our study an increased accumulation of Th17 cells and neutrophils in the CNS of EAE mice was observed. Moreover, the increased production of several cytokines, including IL-17, was detected in the brain during early EAE. Some studies have suggested that IL-17 may induce BBB disruption, thus facilitating the migration of inflammatory cells into the brain [4]. It has also been shown that IL-17A-induced BBB disruption involves the formation of reactive oxygen species (ROS), which are subsequently responsible for reduced expression of tight junction molecules and the deactivation of the endothelial contractile machinery [4].

IL-17 can also interact directly with astrocytes and microglia through IL-17R receptors located on these cells [17]. IL-17A deficient mice display a significantly milder disease and a significantlossof encephalitogeniccapacityafteradoptive transfer of *in vitro* expanded T cells [18]. These data may indicate that IL-17 is important for chemokine expression and development of neuroinflammation in EAE [19]. The increased production of chemokines attracts other inflammatory cells including neutrophils, in line with our observation after stereotaxic intracerebral IL-17 delivery to the brain. We have observed substantial neutrophil inflow to the brain at 72 h after cytokine injection. This may suggest that IL-17 interactions with other cell subpopulations are delayed, which would lead to the production of attractants for neutrophils and stimulate their migration at later time points. As Th17 cells are the main producers of IL-17, its external administration to the brain postponed the accumulation of this T cell subpopulation in EAE brain.

An important determinant of T cell differentiation into Th17 cells is the cytokine IL-23 [20], which was also upregulated in our study in the brain of mice with EAE. IL-23 promotes the development and expansion of activated CD4+ T cells that produce IL-17 upon antigen-specific stimulation [21]. Genetic analysis of these helper T cells identified a unique expression pattern of proinflammatory cytokines and other novel factors. IL-17 expression was undetectable in the CD4+ T cells of IL-23 - deficient mice (derived from either CNS or lymph nodes), suggesting that IL-23 is essential for the development of specific IL-17- producing T cells [22]. It was shown that blocking IL-23 function can alleviate EAE symptoms. Moreover, administration of anti-IL-23p19 antibodies reduced IL-17 levels in the CNS as well as the expression of IFN-gamma, IP-10, IL-17, IL-6, and TNF-alpha mRNA [23]. Our experiment modulating the course of EAE with monoclonal blocking antibodies against IL-23R confirms this hypothesis. In that experiment the first EAE symptoms were delayed and severity of the disease was decreased in the group of animals treated with the anti-IL-23R antibody. The first signs of EAE were seen 4 days later than in the control group and the average clinical scores were significantly lower. Blocking the IL-23R receptor by a monoclonal antibody also significantly reduced the Th17 cell accumulation in the brain. This observation supports the concept that a blockade of this receptor has a direct influence on the differentiation of Th17 cells.

The chemokine receptor CCR6 plays an essential role in the initiation of EAE by controlling the migration of the first wave of autoreactive Th17 cells into the normal CNS. The entry of CCR6+ T cells into the CNS most likely occurs through the blood-cerebrospinal fluid barrier, as epithelial cells of the choroid plexus constitutively express the CCR6 ligand, CCL20. This first wave of hematogenous T cells initiates the recruitment of the second wave of inflammatory T cells that enter the CNS parenchyma in a CCR6-independent manner through the activated parenchymal postcapillary venules [24]. In this context, we reported the increased production of CCL20 in the CNS during EAE attack. As previously reported, CCL20 is highly expressed in the CNS during EAE [25], suggesting that CCR6 is expressed on Th17 cells and neutrophils. As CCR6+ Th17 cells exhibit a strong chemotaxis toward CCL20, a positive feedback may occur wherein Th17 cells further recruit other CCR6-expressing T cells to the site of inflammation [26].

It has been suggested that IL-17 interacts with plurality of inflammatory cells by stimulating expression of CXC ELR(+) chemokines such as CXCL1 and CXCL2, which display a strong chemotactic activity towards neutrophils [27]. Therefore, a growing amount of evidence confirms the crucial role of Th17 cells in the pathogenesis of EAE and MS and justifies the assumption that neutrophils may greatly contribute to the development of the disease. Indeed, CXCL1 has been shown to be the most highly expressed ligand for CXCR2 in the brains of mice subjected to EAE [28].

Under our conditions, however, stereotactic intracerebral microinjection of CXCL1 resulted in neutrophil infiltration into the CNS but did not reveal the presence of Th17 cells directly after delivery. IL-17 produced by Th17 cells affects the production of other inflammatory chemokines and cytokines responsible for EAE development. This might suggest that administration of CXCL1 to the brain may act directly on neutrophils bypassing the stage of IL-17 activation. It has also been shown that the inflammatory process in EAE is strongly correlated with CXCL1 expression [29, 30], and McColl et al. showed that the elimination of circulating neutrophils stimulates resistance to EAE induction [31]. This observation was confirmed by Carlson et al., who demonstrated that depletion of neutrophils in mice protects them against EAE development and this protective effect expires immediately upon reconstitution of circulating granulocytes [7]. Moreover, they found that CXCR2 knockout mice did not develop EAE [32]: but that the transfer of neutrophils expressing this receptor into these mice revoked their resistance to the disease. These data suggest the existence of a pathogenic pathway leading from Th17 cells to neutrophils, via ELR CXC chemokines(+), and highlight the crucial role of these interactions in the development of EAE and MS. However, blocking the granulocyte receptor CXCR2 can decrease demyelinated lesions while enhancing remyelination in EAE mice, as confirmed by the experiments of Liu et al. and Kerstetter et al. [32, 33].

Neutrophils can induce Th17 cells migration by CCL2 and CCL20 expression; however, Th17 cells interact with neutrophils via both an IL-17-dependent and an IL-17-independent pathway. This first pathway leads to neutrophil migration to inflammation sites by the induction of CXCL1 and CXCL2 expression, which are neutrophil chemoattractants. The second proposed pathway is based on Th17 cell production of GM-CSF, TNF-*α*, and IFN-*γ*, leading to the recruitment and activation of neutrophils [16]. As such, blocking CXCR2 on neutrophils does not necessarily lead to a noticeable reduction of neutrophils in the brain during EAE or alter the course of the disease, precisely because of the presence of the second pathway.

We also observed that CXCR2 is expressed on Th17 cells and neutrophils from the brain. Interestingly, administration of anti-CXCR2 monoclonal antibodies to immunized mice did not influence the migration of Th17 cells to the brain. This could be explained by the complexity of chemoattracting signals and the involvement of other chemokines in the regulation of cell migration and retention in brain tissues. However, our results also suggest a minor role of CXCR2-dependent attraction of effector lymphocytes at this stage of inflammation. Perhaps CXCL1 and CXCL2 can induce neutrophils, but not CXCR2+Th17 cells, to migrate into the site of inflammation.

The chemokine CXCL16 and its receptor, CXCR6, are also very important mediators of EAE development and Th17 cell interactions with neutrophils. CXCL16 is expressed by antigen-presenting cells such as monocytes, macrophages, B cells, and dendritic cells [34]. We found high levels of this chemokine in mouse brains, and moreover neutrophils demonstrated an increased expression of CXCR6. The elevated CXCL16 production in the EAE brain may help to recruit CXCR6+ cells across the BBB into gray matter foci, but additional, unknown signals provided during injury are required for gray matter infiltration [35]. It has been shown that administration of neutralizing antibodies against CXCR6 reduced EAE severity and inflammatory cell infiltration of the CNS [36].

In summary, we characterized the chemokine receptor profile on Th17 cells and neutrophils accumulating in the CNS during early EAE and identified some of the interactions mediated by chemokines between these cell subpopulations during development in this MS model. Our data suggest that Th17 cells, neutrophils, and some chemokines could be promising targets for future MS therapies.
